# Predictors of reading comprehension and profiling of poor readers in Croatian: educational and clinical perspectives

**DOI:** 10.3389/fpsyg.2024.1297183

**Published:** 2024-05-14

**Authors:** Jelena Kuvač Kraljević, Nikolina Runje, Valentina Ružić, Ana Matić Škorić, Mirjana Lenček, Antonia Štefanec

**Affiliations:** ^1^Department of Speech and Language Pathology, Faculty of Education and Rehabilitation Sciences, University of Zagreb, Zagreb, Croatia; ^2^Naklada Slap, Centre for Education and Research, Jastrebarsko, Croatia

**Keywords:** decoding, language comprehension, reading comprehension, Simple View of Reading, poor decoding skills, poor comprehension skills, mixed reading difficulty

## Abstract

**Introduction:**

Reading is an important academic skill. Children who exhibit reading difficulties are more likely to experience various negative professional and personal consequences. To successfully identify children with reading problems as early as possible, one must first understand how reading skills can be mastered, as well as the course of reading development in children with typical reading skills from the beginning of their formal reading instruction. Thus, the purpose of this study was to determine the influence of decoding and language comprehension on reading comprehension ability. In addition, this study aimed to determine types of profiles among poor readers and estimate their frequency in the study sample.

**Methods:**

Based on the Simple View of Reading model, we developed decoding, language comprehension, and reading comprehension tasks. Participants included 100 typical readers (TR) and 95 poor readers (PR) in the second grade in Croatian schools. Phonemic awareness, phonological working memory, and rapid automatized naming tasks were used to test underlying abilities of decoding skills in both groups of participants.

**Results:**

As expected, PRs showed significantly lower performance on all variables than TRs. The correlations between decoding, language comprehension and reading comprehension are significant in the PR group. The linear regression analysis showed that language comprehension was a significant predictor of reading comprehension for TRs, while decoding and language comprehension were significant predictors of reading comprehension for PRs. The profiling of reading difficulties revealed five different profiles, the most common of which was a mixed reading difficulty, i.e., difficulties in both decoding and language comprehension.

**Discussion:**

In line with theoretical expectations, success in reading comprehension in TRs at the end of the second grade depends mainly on language comprehension. To achieve this complex cognitive skill, PRs‘ language comprehension alone is not sufficient, so they still rely on their decoding skills. Among the poor readers, there was a high prevalence of children with decoding problems (i.e., three out of five profiles). Teachers should be able to identify and monitor decoding difficulties in children, since these difficulties are associated with noticeable manifestations, unlike those associated with comprehension difficulties.

## Introduction

A large amount of empirical evidence on reading development has been collected over the last 20 years mainly attempting to define similarities and differences in the reading development of different languages depending on their orthographic characteristics (Zaretsky et al., 2009; Ziegler et al., 2010; Florit and Cain, 2011; Landerl et al., 2013; Torppa et al., 2016). One of the general conclusions from these findings is, for example, that transparent orthographies that show a high degree of correspondence between graphemes and phonemes (such as those in Finnish, Italian, or Croatian) are acquired more easily and rapidly than opaque or non-transparent orthographies that are characterized by inconsistent and irregular spellings (such as in English). However, it is important to emphasize that the data collected in one language cannot be directly transferred to another language, even between languages that have similar orthographic characteristics.

Therefore, purpose of the present study is to provide further evidence on the role and contribution of proximal processes – decoding and language comprehension - to the reading comprehension abilities of children in the Croatian language. Croatian is a language with a transparent orthography that, however, differs from the transparency of orthography in Finnish or Italian. In order to define the specifics of reading development based on orthography, as well as similarities and differences between the languages with transparent orthography, several important aspects must be considered (Florit and Cain, 2011; Torppa et al., 2016): (i) the use of a reading model that has already been tested in several languages other than English in order to be able to compare data; (ii) the inclusion of languages with transparent orthography that differ in their transparency; (iii) the need to analyze reading skills in different years of exposure to reading instruction; and (iv) the need to examine the above-mentioned factors in relation to children with reading disorders (Catts et al., 2006). Our study is based on the theoretical framework outlined by the Simple View of Reading model (SVR; Gough and Tunmer, 1986), one of the most well-known reading models that has been studied in different languages. We aimed to assess the reading skills of second-grade children with typical reading abilities, as well as those who experienced reading difficulties.

### Simple View of Reading: theoretical and clinical considerations

The SVR model considers reading comprehension to be a product of decoding and language comprehension, both of which are equally important (Gough and Tunmer, 1986; Hoover and Gough, 1990). Decoding skills translate print into language and the comprehension component helps make sense of the linguistic information (Catts et al., 2006). Decoding skills can improve word recognition and this is supported via two different routes (Coltheart and Rastle, 1994): phonological - in which a word is recognized on the grapheme-phoneme basis, and orthographic - in which well-known words are recognized automatically. The activation of these two routes depends on orthographic transparency, reading proficiency, as well as the phonological, morphological, and semantic complexity of the words in the text that is being read (Kirby and Savage, 2008).

It is clear that these two routes conceal numerous cognitive processes and abilities. For example, phonological decoding through the phonological route depends heavily on phonological awareness and the functionality of the phonological working memory, i.e., phonological information must be stored temporarily, while the reader connects the series of phonemes and graphemes (Wagner and Torgesen, 1987). Orthographic processing depends on rapid automatized naming (RAN), i.e., the cognitive ability to retrieve lexical items quickly to recognize words, as well as on a number of other factors that are more environmentally conditioned, such as length of exposure to written language or methods of reading instruction (Kirby and Savage, 2008; Torppa et al., 2016; Sánchez-Vincitore et al., 2022).

Owing to its comprehensibility, the SVR has proven to be a valid model for reading comprehension in many languages. Research shows that, on average, the SVR accounts for 40–60% of the variance in reading comprehension (Foorman and Petscher, 2018). In transparent languages, this proportion is even higher. For example, in a Dominican Spanish study, Sánchez-Vincitore et al. (2022) found that word recognition and language comprehension explained 80% of the variance in reading comprehension after 4 years of reading instruction.

The impact of decoding and language comprehension on reading comprehension varies across grades, i.e., the child’s reading ability. Because beginning readers start to learn to read with some degree of language comprehension, decoding, rather than language comprehension, should have the greatest influence on reading comprehension in the early grades (Torppa et al., 2016). When word recognition is relatively fast and automatic, more of these processing resources can be devoted to reading comprehension. Therefore, in later years, language comprehension plays a more significant role in predicting reading achievement. In Florit and Cain (2011) meta-analysis, it was shown that, for transparent orthographies, language comprehension has a stronger influence on reading comprehension than decoding, even for beginning readers.

Although the SVR was developed primarily for educational use, it has developed an increasing clinical significance. From the beginning of its development, the SVR has constantly emphasized that both basic components are necessary for success in reading and that neither is sufficient by itself. According to Gough and Tunmer (1986), reading comprehension difficulties may be caused by decoding difficulties, an inability to comprehend language, or both of these skills. Accordingly, three clinical conditions associated with reading difficulties can be distinguished. Children who exhibit decoding difficulties, but do not have language comprehension difficulties are classified as having dyslexia. This condition is generally associated with problems in the phonological domain, especially with difficulties in phonemic awareness and phonological recoding, which ensures consolidation of orthographic representations in lexical memory (Tunmer and Greaney, 2009). The mixed difficulty group has both decoding and language comprehension difficulties. In addition to phonological processing deficits, these children have more widespread language impairments – in vocabulary, morphology, syntax, and/or discourse-level processing – that can influence text comprehension. In their longitudinal study, Tunmer and Chapman (2007) found that, in addition to the expected differences on oral language measures, readers with mixed difficulties showed consistently greater phonological processing deficits than readers with dyslexia across a range of phonological processing measures. Finally, children who show difficulties in language comprehension in the absence of decoding difficulties are classified as readers with poor comprehension skills. These poor comprehenders are generally free of phonological processing deficiencies and demonstrate satisfactory alphabetic coding skills, but show weaknesses in vocabulary, morphology, syntax, discourse-level processing, and/or comprehension strategies, which in turn negatively affect reading comprehension performance (Tunmer and Greaney, 2009).

In a study involving a cohort of 183 poor readers in the second grade, Catts et al. (2003) reported that 35% had dyslexia and 35% mixed reading disabilities. According to Tunmer and Greaney (2009), the most common reading profile is the one with deficits in both components – decoding and language comprehension, i.e., mixed reading disabilities. According to Torppa et al. (2007), the prevalence of reading difficulty subtypes varies greatly with age and sample size. Based on the SVR and an advanced mixture modeling procedure for two latent factors, one for word recognition fluency and one for reading comprehension and their covariance, Torppa et al. (2007) categorized the reading profiles of 1750 children at four measurement time points in the first 2 years of their schooling. The authors identified five different subtypes of reading profiles: (1) poor readers (poor word recognition and reading comprehension skills), (2) slow decoders (poor word recognition fluency combined with reading comprehension that reached an average level over time), (3) poor comprehenders (average word recognition combined with a time delay in reading comprehension), (4) average readers (average word recognition and reading comprehension), and (5) good readers (above average word recognition and reading comprehension). Interestingly, the same authors emphasized that identification of reading difficulties in languages with transparent orthography must include measures of reading fluency, because beginning readers acquire basic reading accuracy relatively easily and quickly (Torppa et al., 2007).

The present study strives to define the role of decoding and listening comprehension on reading comprehension in Croatian by analyzing the reading skills of children in the second grade, i.e., after 2 years of formal reading instruction, both typical readers and readers who are struggling with reading. Following Ehri (2002) who classified the phases of learning to read, this period corresponds to the consolidated alphabetic phase, in which children read using orthographic mapping through phoneme-grapheme linkages, word families, syllables, and morpheme patterns (Beech, 2005).

### Croatian educational system

The Croatian language belongs to the Slavic group of languages. Typologically, it is a highly inflected, pro-drop language. The Croatian alphabet has 30 letters and 32 phonemes (with vowel r and diphthong ie) (Barić et al., 2005). Three graphemes are digraphs (*lj*, *nj*, and *dž*), which means that one sound is represented by two letters. Learning to read and write in Croatian is very similar to learning to read and write in Finnish, Greek, or Italian, because, similar to those languages, there is a high proportion of grapheme-phoneme correspondence. In Croatian, both sounds - vowels and consonants - are pronounced in a completely consistent manner, which ensures easy transfer of sounds from the spoken to the written mode. There are only a few inconsistencies (e.g., the spelling of the sonant *j* between two vowels in pronunciation – *avijon*, instead of *avion* (eng., *aeroplane*) - or the sonoric sequence principle - an alternation of voices in which two differently sounding consonants are placed next to each other and the first consonant must be replaced by its (voiced or unvoiced) counterpart (e.g., *precjednik*, instead of *predsjednik*, eng. *president*). These inconsistencies are formally taught in the lower grades of elementary school. If letter-phoneme entropies were applied, the impact of these exceptional pronunciations is rather marginal in Croatian. According to Borgwaldt et al. (2004); p. 213), …*If a letter always corresponds to one phoneme, then its entropy will be zero, as its pronunciation is completely predictable. The more alternative pronunciations a letter has, the higher its entropy value is…* Borgwaldt et al. (2004) analyzed word-initial letter-to phoneme and phoneme-to-letter ambiguity in five languages (Dutch, English, French, German, and Hungarian) and defined English as the most ambiguous, and Hungarian as a language with the most predictable orthography. If we observe the initial phoneme, the entropy value in Croatian would be zero, but if we focus on the middle position, where the above-mentioned inconsistencies occur, the entropy in a very small number of words would be 2. In general, the entropy value for Croatian is low, which means that spelling can be predicted largely from pronunciation. However, due to the few inconsistencies mentioned above, if we were to compare Croatian to some other languages with transparent orthographies, it is somewhat less transparent than Finnish and more similar to Italian (for entropy values in other languages, see Borgwaldt et al., 2004 and Ziegler et al., 2010).

Compulsory primary education begins at the age of 7 years. However, the level of reading and writing readiness varies significantly among students. Preschool programs are not uniformly defined, especially in terms of expected levels of early literacy and, more importantly, language preparation (Lenček and Užarević, 2016; Kuvač Kraljević et al., 2019). In the first 2 years of school, literacy is predominantly taught as part of the school subject “Hrvatski jezik” (Croatian language), in which reading, writing, and other native language topics are taught for a total of 175 instructional hours per year, with one instructional hour corresponding to 45 min. Budinski (2019) pointed out that Croatia is among the European countries with the lowest proportion of total teaching time devoted to the mother tongue (22.7% Croatian - compared to 24% in England, 27% in Slovenia, or 40% in Hungary).

In the first year of school, students master reading and writing in printed uppercase letters, while printed lowercase letters occur rarely in the reading materials. According to the Croatian language curriculum (as prescribed in the Croatian Official Gazette, 2019), by the end of the first year of school, every student should be able to decode a short text and this process should be almost automated. The teaching methods used are global, analytical, and synthetic methods. In the second school year, the student should strive for mastery of upper- and lower-case cursive letters, especially in writing. By the end of this period, the student is expected to read short texts and answer questions about them independently.

Unlike some other countries, such as Estonia and Finland (Soodla et al., 2018), where special support (such as remedial teaching during or after school by class teachers) is provided for children with reading difficulties, regardless of their etiology and without any formal diagnosis, in the Croatian education system, the wait-to-fail model is still the dominant model. This means that children with reading difficulties are usually not considered until the third grade. A formal diagnosis is required for these children to receive any kind of school support and this diagnosis can only be established outside the education system.

In general, there is a relatively small number of studies that describe the characteristics of reading in Croatian (Lenček and Ivšac, 2007; Kelić, 2019; Kelić et al., 2021). Most studies have focused on describing the characteristics of reading in children with dyslexia. For example, Vancaš (1999) showed that in decoding pseudoword lists, lists saturated with letters characteristic for Croatian Latin script (*č, ć, dž, đ, lj, nj, š, ž*) were difficult for typical children and even more difficult for children with dyslexia. Lenček and Anđel (2011) point that that children with dyslexia in Croatian produce all kinds of mistakes during reading known from the dyslexia related literature: substitutions (in particular of pairs *b*-*d*-*p*; *m*-*n*) as the most frequent type of error, then additions of phonemes (mostly vowels), and, finally, the least frequent - omissions. The only study (Kelić et al., 2021) that aimed to define the role of reading predictors showed that after 3 years of formal reading instruction i.e., when reading is automatized, RAN is the most significant predictor of both reading accuracy and reading speed. In addition, the study confirmed the importance of phonemic awareness as a suppressor variable for RAN in predicting pseudowords reading time.

The development of reading data in neighboring languages that are typologically and orthographically similar to Croatian, such as Slovene and Bosnian, and on whose descriptions, we could rely in the absence of one’s own descriptions, has also hardly been researched. There is little data on reading in Bosnian, which is also mainly focused on describing the characteristics of reading in children with dyslexia (Duranović, 2017; Duranović et al., 2018). This situation is related to the fact that research efforts in all these Slavic languages that use Latin alphabet and have transparent orthography started recently i.e., about 20–30 years ago.

### Present study

The aim of this study was to examine the predictors of reading comprehension in Croatian by analyzing reading skill of children who had almost completed the second grade. This is the period in which formal reading instruction has ended and the expectations of the education system are that the child will start to use reading as a tool for further learning.

The specific research questions of this study are:

1) How do typical and poor readers perform on measures of accuracy and speed on various reading tasks, as well as underlying cognitive reading skills after 2 years of formal instruction?
2) How predictive are decoding and language comprehension for reading comprehension at the end of the second grade for typical and poor readers?
3) Taking into account the performance of poor readers in relation to decoding and language comprehension, how many profiles of reading difficulties can we identify and which of these are the most common? Apart from decoding and language comprehension, what other underlying skills can define reading difficulties?

The present study addresses these research questions based on two perspectives: educational, in order to define the predictors of reading that precede successful reading comprehension; and clinical, in an attempt to define subtle differences in decoding and language comprehension in children with reading difficulties.

## Methods

### Participants

The study sample consisted of a total of 195 children i.e., 100 typical readers (TRs) and 95 poor readers (PRs). Sample was stratified by gender and region, in accordance with Census of the Croatian Bureau of Statistics (2011). All participants were attending the 2nd grade for the first time, spoke Croatian as their first language, and had normal motor skills, as well as visual and auditory processing abilities, as confirmed by information in children’s school files. The average age of the TRs was 8.07 years, while the average age of the children with reading difficulties (i.e., PRs) was 8.10 years. There was no statistically significant difference in the proportion of male and female participants in TR and PR groups (χ2 (1, 195) = 0.642, *p* = 0.423).

A total of 42 speech and language pathologists (SLPs) from 11 different schools and 8 different clinical institutions from different parts of the Republic of Croatia participated in the study as examiners. All examiners were trained on how to collect data by the research team before the start of the study.

All SLPs were instructed to carefully follow inclusion and exclusion criteria when recruiting both TRs and PRs. SLPs working in primary schools were advised to consult school files and other available information about the child’s functioning in order to gain insights into their cognitive and emotional functioning, as well as their academic skills. They were also instructed to obtain further information about the children from parents and second grade teachers, whenever possible. SLPs working in clinical institutions had to consider assessment results of both SLPs and psychologists when recruiting children with reading difficulties. Considering the PRs, it was important that there was a history of either reading or language difficulties, as well as a history of receiving learning support and/or therapy, and no evidence of cognitive and emotional difficulties. Supplementary Appendix Table 1 lists the inclusion and exclusion criteria for both TRs and PRs. All the engaged SLPs were instructed on how to recruit children and advised to carefully read the inclusion and exclusion criteria before giving out informed consent documents to parents and children.

Basic information about participants such as age, sex, previous involvement in a preschool program, regular school enrolment, and learning support were also collected and analyzed (Table 1). In addition, information about the education levels of the parents of the included participants was also collected (Table 2).

**TABLE 1:** Participant data.

	*n*	Age (*M*/*SD*) in months	Sex (F/M)	Attending preschool (%)	Regular school enrolment (%)	Learning support (%)
2^nd^ grade Typical readers	100	103.33/3.511	49/51	85.0	92.0	0.0
2^nd^ grade Poor readers	95	106.16/5.565	52/43	81.1	74.7	40.0

**TABLE 2:** Education levels of the parents of children included in the study.

	*n*	Primary school graduate and lower (%)	High school graduate (%)	Bachelor’s degree (%)	Master degree and higher (%)
**Mother’s education level**					
2^nd^ grade Typical readers	99	2.0	41.4	20.2	36.4
2^nd^ grade Poor readers	93	10.8	51.6	18.3	19.4
**Father’s education level**					
2^nd^ grade Typical readers	98	7.1	56.1	12.2	24.5
2^nd^ grade Poor readers	93	9.7	64.5	14.0	11.8

For some children included in the study, data about parental education is missing. Therefore, data reported in this table are smaller than the overall sample.

Overall, the data shows that the education levels of the parents follow the educational trend in Croatia (according to the last census, Croatian Bureau of Statistics, 2011), where high school graduates are the most common. However, mothers of TR children differed significantly regarding education levels from mothers of PR children (χ2 (3, 192) = 11.951, *p* < 0.01), indicating that a higher proportion of children in the TR group had mothers with higher education than children in the PR group (and vice-versa, proportion of lower educated mothers was higher in the PR group). On the other hand, there was no statistically significant difference in the proportion of education of fathers between the two groups of children (χ2 (3, 191) = 5.209, *p* = 0.157).

### Materials

In order to develop valid test material, a Corpus of written language at school age was created (Riddys; Kuvač Kraljević and Lenček, 2020) comprising of selected textbooks for the lower grades of elementary school. The corpus includes a total of 502,713 tokens and about 45,000 sentences categorized into four subcorpora based on grade. All four subcorpora were lemmatized and marked both morphosyntactically and syntactically using ReLDIanno (Ljubešić and Erjavec, 2016).

The Corpus was analyzed at phonological, morphological, and syntactic levels. Through this approach, data were obtained on the phonemic structure of words (length, phonological complexity, syllable structure, bigram frequencies) that children are typically exposed to in written language. Morphological analysis was used to classify words into grammatical types according to their morphosyntactic features, and syntactic analysis provided data on the total number of sentences, as well as the type and average length of sentences. These data served as empirical information on what children experience in the first 4 years of elementary school in terms of written language (see more in Matić Škorić et al., 2023). Taken together, this knowledge served as a linguistic starting point for the creation of the items in the test material.

**Phonenic awareness:** Phonemic awareness was assessed using two tasks: firstly, phoneme blending and segmentation and secondly, phoneme deletion and addition. The first group of tasks - phoneme blending and segmentation – consists of two subtasks (blending and segmentation) with 5 items each. The second group of tasks – deleting and adding – consist of four tasks: phoneme-deleting task on first phoneme in word, phoneme-deleting task on last phoneme in word, phoneme-adding task on first phoneme in the word, and phoneme-adding task on last phoneme in the word - each with 5 items. For example, children were presented with a word /*krasti*/ and asked to delete the first sound /*k*/ and say the remaining sequence /*rasti*/ out loud. Or, they were asked to add the last sound /*v*/ on a word /*ruka*/ and to say the remaining sequence /*rukav*/ out loud. All words were controlled in length (from monosyllabic to pentasyllabic) and phonological complexity.

Since the tasks of blending and segmentation represent a lower level of phonemic awareness, i.e., it is acquired earlier and more quickly, this level was observed separately from the ability to delete and add phonemes, which represents a higher level of phoneme manipulation. Therefore, two unique variables were analyzed - phonemic awareness: blending/segmentation (max = 10) and phonemic awareness: deleting/adding (max = 20). Cronbach’s alpha for a phonemic blending and segmentation task was 0.754 and for a deleting and adding task was 0.744.

**Phonological working memory:** The nonword repetition task was used as a measure of phonological memory. In this task, students were required to repeat eight nonwords, ranging from one to six syllables in length. The dependent variable was the number of correctly repeated pseudowords (max = 8). Cronbach’s alpha for this task was 0.727.

**Rapid automatized naming:** RAN was assessed based on a standard procedure described in Denckla and Rudel (1976), in which children were asked to name a series of five objects as quickly as possible (for example, *cheese, glass, hedgehog, key, table* – *sir, čaša, jež, ključ, stol* in Croatian) arranged in semi-randomized order in five rows of five. The test was preceded by a practice trial to ensure that each child was familiar with the objects. The total time for naming all stimuli served as the child’s score. There were few errors in object naming accuracy, and therefore naming accuracy was not considered in further analysis.

**Decoding:** A word fluency and a pseudoword fluency task were administered to assess decoding skills. Therefore, children were presented with two lists, each consisting of 17 items. Reading accuracy and speed were assessed.

On the basis of the RiDDys corpus, the word list contained high-frequency words that were expected to be orthographically processed. The lists consisted of two- to five-syllable words with varying phonological complexity. In addition, the words were written in their basic morphological form (nominative for nouns and infinitive for verbs).

The list of pseudowords was created using an approach where subsyllabic elements were combined (König et al., 2020), in a way that the criteria of length, segmental complexity, phonotactic probability, accent system, and wordlikeness were controlled when creating the pseudowords [the whole process of pseudoword creation was explained in detail in Kuvač Kraljević et al. (2022)]. Accordingly, a list of orthographically legal and pronounceable pseudowords was created.

Each correctly read word and pseudoword was assigned a score of 1 point (max = 34). Cronbach’s alpha for this task was 0.746.

**Language comprehension:** According to Sánchez-Vincitore et al. (2022), language comprehension within the SVR can be tested through vocabulary, morphosyntactic ability, and oral discourse comprehension tests. Since discourse level integrates various language skills and knowledge, we decided to use it as a measure of language comprehension. Four short stories were presented orally to the children. Three stories consisted of only two sentences, and one consisted of four sentences. These four stories were designed to elicit different types of questions, such as literal, cohesive, vocabulary, and knowledge-based questions [question classification is described in Bowyer-Crane and Snowling (2005)]. For example,

*A boy from the neighboring street chased the wasps away from the bench where the girls were sitting. He saved them from being stung.*

a) *Is the boy fearless?*
b) *Did the wasps sting the little girls?*
c) *Can the boy be proud?*

This story has two vocabulary-based questions - the children must know the words fearless and proud to give the answers - and one evaluative question - did the wasps sting the little girls? – this question can only be answered if the children were able to understand the last sentence. Needless to say, the children must follow the story, temporarily retain the information, and understand the event and the relationship between the actors to give one of three possible answers – yes, no, or I do not know. After each story, the children gave answers to three different questions and 1 point was awarded for each correct answer (max = 12). Cronbach’s alpha for this task was 0.674.

**Reading comprehension:** This skill was tested using two tasks. In the first task, children were presented with a culturally appropriate story consisting of 12 sentences and 90 words and they were asked to read the connected text. In this task, both components of fluent reading – accuracy and speed – were assessed. The score corresponded to the number of words read correctly and the time taken to read the text. After reading a story, the child had to answer reading comprehension questions: eight literal comprehension questions, six of which were open-ended, but required a short answer of one or two words, and two of which were based on multiple-choice questions. 1 point was awarded for each correct answer (max = 8). Cronbach’s alpha for this task was 0.690.

In the second task, children were exposed to 12 short stories – each with two sentences, except one that had three sentences – and they were expected to respond with answers based on multiple-choice tasks. The children were expected to read these stories silently because the main goal of this task was to assess comprehension of what is being read and not reading fluency. The stories were chosen so that they elicited literal, cohesive, vocabulary, and knowledge-based questions (Bowyer-Crane and Snowling, 2005). Here is an example of a cohesive task:

*They did not stop talking after his warning. Who warned them?*

a) *they*
b) *she*
c) *he*
d) *me*

Each correct answer was scored with 1 point (max = 12). Cronbach’s alpha for this task was 0.714. As both tasks measure the same ability - reading comprehension - they were further analyzed together.

### Procedure

A total of 195 participants were recruited as part of a research project entitled *Development of an innovative diagnostic instrument for early recognition of children with dyslexia (RiDDys)* (KK.01.2.1.02.0167). This project was developed within the framework of the funding program “Increasing the development of new products and services arising from R&D activities – Phase II” (IRI 2), the goal of which was to develop the first standardized test for diagnosing reading disorders in Croatia. SLPs working in schools and clinical settings (public or private) were contacted and requested to help with data collection. They were instructed to select participants based on the criteria outlined by the research team (see list of criteria in Supplementary Appendix Table 1). In order to stratify the sample of participants by region, SLPs were contacted based on the region where they work.

SLPs who were employed in schools were instructed to randomly select typical second grade students by choosing every second student in the class register. In order to minimize the effect of teaching methods on results of the study, SLPs in primary schools were advised to recruit children from one class. SLPs in schools could also conduct an assessment of the students who were recognized by the teacher as showing difficulties with reading. In this case, both the SLP and the student’s teacher had to report briefly on the nature of the difficulty. SLPs employed in clinical settings (polyclinics, clinics, private practices), who worked almost exclusively with children with language, speech and communication disorders, were instructed to assess children with reading difficulties based on previous SLP and psychological assessments of their reading skills.

Each SLP examined an average of five children and were given sufficient time to perform the assessments. The parents and the children gave their consent for participation by signing the informed consent form. The inclusion of SLPs in the study, the recruitment of participants from the primary schools, and the entire testing procedure was approved by the Ministry of Science and Education of the Republic of Croatia (Class: 602-02/21-01/26; Number: 561-03-01/7-21-2; March 19, 2021).

Each child was assessed individually in a quiet room in May 2022, i.e., a month before the end of the school year. The assessment lasted approximately 60 min. If a child showed signs of fatigue or had to leave to attend a class, the assessment was split into two sessions. The second session was carried no later than 7 days after the first session. All materials were scored and coded by the members of the research team.

### Data analyses

All statistical analyses were performed in IBM SPSS 27. First, descriptive statistics were calculated for each variable for both groups of participants - TRs and PRs. Since the variables did not deviate from the normal distribution, the differences between groups on all variables were tested using parametric statistics, i.e., independent samples *t*-test.

Correlation analysis was conducted, for TRs and PRs separately. Then, a linear regression analysis was performed with reading comprehension as the criterion variable and decoding and language comprehension as predictor variables for TRs and PRs separately.

To determine the specific reading profiles in the PR group, means and standard deviations for decoding and language comprehension were calculated for the TR group which consisted of 100 typical second grade readers who were representative of this population in Croatia. If the child scored −1*SD* or lower than the average score of a TR on any of these two variables, it was concluded that the child exhibited difficulties in that skill. This data was then used to determine the child reading profiles. For each group, we calculated the percentage of children exhibiting different combinations of difficulties: (1) only decoding difficulty, (2) only language comprehension difficulty, (3) both decoding and language comprehension difficulties.

## Results

### Performance of TRs and PRs on measures of accuracy and speed on various reading tasks and underlying cognitive reading skills

To answer the first research question, a *t*-test analysis was performed. Since there was heterogeneity of variance between groups, Welch’s correction for variance heterogeneity was applied and there was no change in significance (all *p* values remained below 0.001). Descriptive statistics of the results and scores obtained from the *t*-test analysis show that TRs were able to achieve significantly better scores than PRs on all tested variables (Table 3). In addition, there was a variability in the results of both groups for all measured variables, i.e., mistakes were made on all tasks, even in the TR group.

**TABLE 3:** Descriptive data on all measured variables for both groups of participants and results of *t*-test analyses.

Variable	Group	N	*M*	*SD*	SEM	df	*t*	*p*
PA-b/s (max. 10)	TR	100	9.27	1.062	0.106	193	5.569	<0.001
	PR	95	7.63	2.733	0.280			
PA-d/a (max. 20)	TR	100	19.21	1.175	0.117	193	6.347	<0.001
	PR	95	16.42	4.227	0.434			
PWM (max. 8)	TR	100	6.52	1.283	0.128	193	8.509	<0.001
	PR	95	4.63	1.787	0.183			
RAN (in sec)	TR	100	16.33	3.465	0.347	193	- 6.955	<0.001
	PR	95	20.82	5.384	0.552			
Dec-words (max = 17)	TR	100	14.93	2.109	0.211	193	10.172	<0.001
	PR	95	10.14	4.186	0.429			
Dec-pseudo (max = 17)	TR	100	11.82	3.347	0.335	193	9.672	<0.001
	PR	95	6.73	3.993	0.410			
Dec-sum (max. 34)	TR	100	26.75	4.929	0.493	193	10.778	<0.001
	PR	95	16.86	7.654	0.785			
W-read. speed (in sec)	TR	100	37.54	19.086	1.909	192	16.291	<0.001
	PR	93	70.82	58.171	6.032			
PW-read. speed (in sec)	TR	100	60.70	27.724	2.772	193	14.433	<0.001
	PR	94	104.28	105.496	10.881			
LC (max. 12)	TR	100	11.32	0.942	0.094	193	5.778	<0.001
	PR	95	10.22	1.639	0.168			
RC (max 20)	TR	100	18.06	2.988	0.299	191	9.426	<0.001
	PR	95	13.33	3.943	0.409			
Text - read. speed (in sec)	TR	100	77.67	33.045	3.305	193	15.744	<0.001
	PR	94	155.98	131.829	13.597			

PA-b/s, Phonemic awareness - blending and segmentation; PA-d/a, Phonemic awareness - deleting and adding; PWM, Phonological working memory; RAN, Rapid automatized naming; Dec-words, Decoding of words; Dec-pseudo, Decoding of pseudowords; Dec-sum, Decoding of words and pseudowords; W-read. speed, Word list reading speed; PW-read. speed, Pseudowords list reading speed; LC, Language comprehension; RC, Reading comprehension; Text-read. speed, Text reading speed; TR - Typical readers; PR - Poor readers; M - mean; SD - standard deviation; SEM, Standard Error of Mean.

### Contribution of decoding and language comprehension to reading comprehension for TRs and PRs

To answer the second research question, correlation and linear regression analyses were performed for the two groups of participants separately.

The correlation analyses (Table 4) show that the TRs exhibit a weak positive statistically significant correlation only between language comprehension and reading comprehension. It should be noted that the TRs generally had very high average scores and a low dispersion of scores for all the observed measures, as shown in the *M* and *SD* values presented in Table 3. In the PRs, decoding and language comprehension showed a weak positive statistically significant correlation with reading comprehension.

**TABLE 4:** Correlations between decoding, language comprehension and reading comprehension for TRs and for PRs (in brackets).

	Dec-sum	LC	RC
Dec-sum	1		
LC	−0.081 (0.002)	1	
RC	0.115 (0.229*)	0.205* (0.372**)	1

Dec-sum, decoding of words and pseudowords; LC, language comprehension; RC, reading comprehension;

*p < 0.05;

**p < 0.01.

Linear regression analysis was performed to answer research questions about the predictivity of decoding and language comprehension on reading comprehension as the criterion variable for TRs and PRs separately (Table 5).

**TABLE 5:** Linear regression analysis with reading comprehension as criterion variable for TRs and for PRs.

	Reading comprehension of TRs		Reading comprehension of PRs	
Predictor variables	β	*p*	β	*p*
Dec-sum	0.132	0.184	0.227	0.019*
LC	0.216	0.031*	0.371	0.001**
***F***	3.063		10.546	
***p***	0.051		<0.001	
**R^2^**	0.059		0.190	

Dec-sum, decoding of words and pseudowords; LC, language comprehension.

*p < 0.05;

**p < 0.01.

Results for TRs showed that only language comprehension was a significant predictor of reading comprehension. Results for the PRs showed that decoding and language comprehension were significant predictors of reading comprehension and explained 19% of the variance in reading comprehension.

### Profiles of reading difficulties based on performance in decoding and language comprehension, as well as other underlying skills

Based on the results of decoding and language comprehension, an attempt was made to determine certain profiles of PRs among the study participants. Since at this point there are no standardized measures for decoding and oral discourse comprehension in Croatian it was decided to determine a cut-off point of −1*SD* based on the achievement of 100 TRs for whom it is assumed they represent overall population of second graders based on gender and region. This value was used to determine how many of the PRs have results lower than the cut-off and whether there are notable similarities between their results.

**FIGURE 1:**
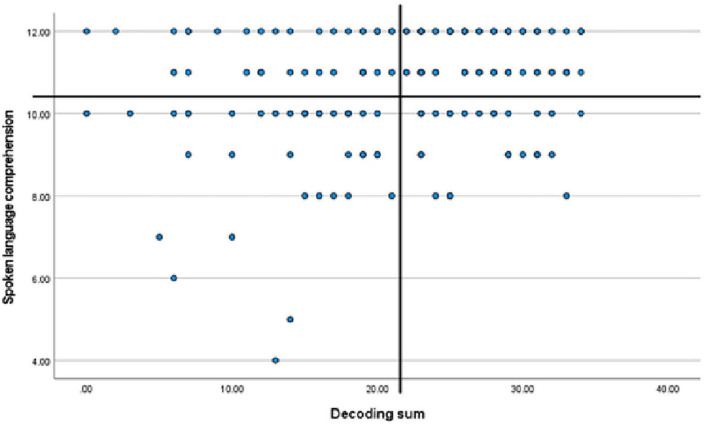
Scatter plot depicting decoding and language comprehension scores of poor readers (*n* = 95) using a cut-off determined at –1*SD* of the mean result of typical readers. Drawn lines represent values at –1*SD* from mean results of typical readers. Dec-sum, decoding; LC, language comprehension.

The cut-off point (*M* - 1*SD* of the results for TRs) was 21.821 points for decoding and 10.378 points for language comprehension. Results of PRs with respect to those cut-off points are shown in Figure 1. If the score on any of these two variables was −1*SD* or lower than the average score of a TR group, it was concluded that the child exhibited difficulty in that skill. The results for each child could fall below average on one of the variables, both of them, or neither.

**FIGURE 2:**
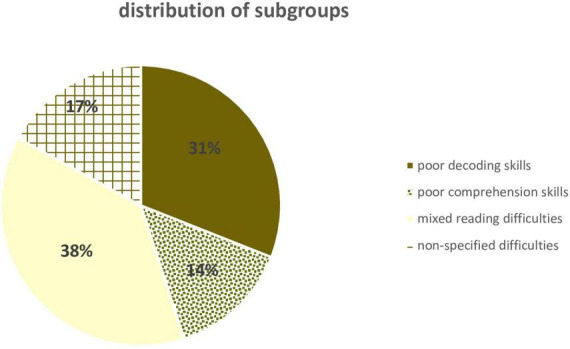
“Distribution of subgroups,” “Poor decoding skills,” “Poor comprehension skills,” “Mixed reading difficulties,” and “Non-specified difficulties.”

The prevalence of each combination of scores i.e., profiles of difficulties for PRs is shown in Figure 2. Results indicate that 31% of PRs in the second grade could be classified as those with lower scores on decoding skills only (poor decoders), 38% as those with lower scores on both measures – decoding and language comprehension (mixed reading difficulties), 14% as those with below average scores on language comprehension (poor comprehension skills), and 17% as having indication for non-specified reading problems (results above cut-off value on both variables but still considered as PRs by speech-language therapists).

Scores of those subgroups of PRs (means and standard deviations), for which a certain type of difficulty is indicated by their results on decoding, language comprehension and reading comprehension are shown in Table 6.

**TABLE 6:** Performance of each subgroup on variable decoding (Dec-sum), language comprehension (LC), and reading comprehension (RC).

Subgroups	Descriptive data	Dec-sum	LC	RC
Poor decoding skills (*n* = 30)	Min-Max	0–20	11–12	9–21
	*M*	12.400	11.566	14.41
	*SD*	6.636	0.504	3.300
	*SEM*	0.663	0.141	0.655
Poor comprehension skills (*n* = 13)	Min-Max	23–31	8–10	10–20
	*M*	26.23	9.153	14.53
	*SD*	2.97	0.800	3.799
	*SEM*	0.932	0.079	0.562
Mixed reading difficulties (*n* = 36)	Min-Max	0–21	4–10	4–20
	*M*	13.416	8.944	11.257
	*SD*	5.373	1.529	4.089
	*SEM*	0.872	0.243	0.697
Non-specified difficulties (*n* = 16)	Min-Max	22–34	11–12	9–19
	*M*	25.375	11.437	14.937
	*SD*	3.095	0.512	3.043
	*SEM*	0.390	0.051	0.338

Dec-sum, decoding of words and pseudowords; LC, language comprehension; RC, reading comprehension.

The group with indication for mixed reading difficulties showed poor performance on reading comprehension, while other groups had similar achievement on this variable. This is consistent with previous studies that have shown that this group had the greatest difficulty in reading, which was due to difficulties in both components - decoding and language comprehension (e.g., Tunmer and Chapman, 2007).

**FIGURE 3:**
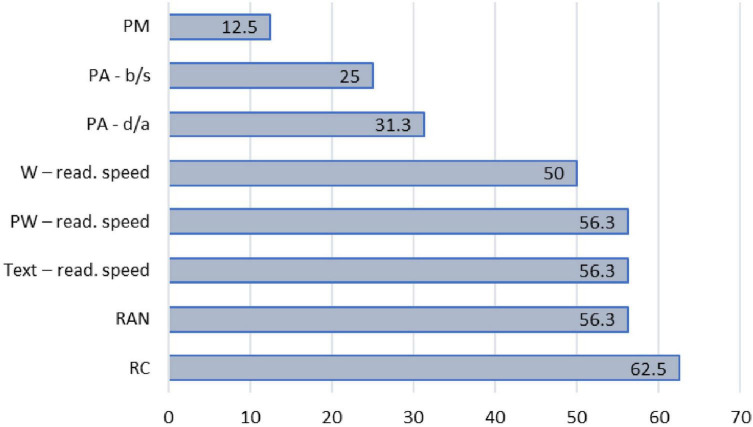
Distribution (%) of variables on which children form non-specified group have a difficulties (*n* = 16). The numbers represent the percentage of children who exhibited a certain difficulty. Since the difficulties usually came in different combinations, i.e., more than one difficulty could be present and the total % exceeds 100%.

The last defined group is with indication for non-specified difficulties. This group is usually mentioned in profiling studies (e.g., Catts et al., 2003), but the details related to the characteristics of children belonging to this group are rarely outlined. In the present study, we decided to extend the current findings by taking a closer look at this group. We started by noting down specific difficulties of each child belonging to this group (Supplementary Appendix Table 2) and then observed the frequency of each difficulty in this group (Figure 3). The same criteria used to define difficulties in decoding and language comprehension were used to define difficulties in phonemic awareness, phonological working memory, rapid automatized naming, as well as word- pseudoword- reading accuracy and text reading speeds. If the child had a score which was −1*SD* or lower than the average score of TRs, they were categorized as having indication of difficulty in that specific ability.

Among the patterns of the results of children with indication of non-specified difficulties, the biggest subgroup of children (*n* = 9, 62.5%) exhibited characteristics similar to a group referred to by Torppa et al. (2007) as slow decoders (poor word recognition fluency combined with reading comprehension). Among them, one child showed problems solely on the text level, accompanied by slower retrieval of phonological information measured with RAN. For the remaining eight children, the main problems were related to reading speed measured on all three levels, i.e., while reading a list of words, pseudowords, and text. For most of them (*n* = 5), this problem was accompanied with reading comprehension difficulties, however, for the rest of them (*n* = 3) this was not the case. Two of these three children did not have any other difficulty whatsoever, whereas one had some problems at the level of phonemic awareness.

The other seven children in the indication of non-specified difficulties group showed a more varied combination of difficulties. For example, one child had problems only while reading a list of words and another exhibited poor reading comprehension abilities. These may either be children who have been monitored, but in principle belong to a group of typical readers, or children who had lower levels of attention or motivation during assessment. Five children exhibited problems with RAN, in combination with either lower or more advanced levels of phonemic awareness, or with phonological memory in a pseudoword repetition task. These seven children form the smallest group, with average scores on most skills and with sporadic difficulties on different individual tasks.

It can be concluded that the children in the group with indication of non-specified difficulties can be roughly categorized into two profiles - first, a somewhat larger group of children that could be considered as slow decoders, and second, a somewhat smaller group, as just described, with average results on most skills and sporadic difficulties on different individual tasks.

## Discussion

The aim of this study was to examine the predictors of reading comprehension in Croatian. More specifically, the goal was to determine how decoding and language comprehension contribute to reading comprehension at the end of the second grade. This grade level represents the end of the initial reading period, which is a 2-year period during which the educational system is focused on reading development. According to the classification of the phases of learning to read as described in Ehri (2002), at the end of this period, children are in the consolidated alphabetic phase, where they slowly start to reach automaticity in their reading ability.

The present study was conducted from two perspectives. The first is educational, since it examines the reading predictors that precede successful reading comprehension. The second perspective is clinical, in which attempts were made to define subtle differences in decoding and language comprehension in children with reading difficulties. An analysis of these differences makes it possible to define different profiles that exist beyond visible reading difficulties.

### Performance of typical and poor readers

Detailed analysis of the results obtained showed that, as expected, the PR group had significantly lower performance on all examined tasks. These data are consistent with many other studies that have examined language and cognitive measures of reading in the early years of formal reading instruction among PRs (Catts et al., 2003; Torppa et al., 2007).

More specifically, our findings show that, at both levels of phonemic awareness (the lower level being phonemic blending and segmentation and the higher level being deleting and adding phonemes), TRs have high scores with very little dispersion of results and minimal errors. These high scores are consistent with previous data on early mastery of phonemic awareness in languages with transparent orthography in school, as found in studies by Ziegler et al. (2010) and Torppa et al. (2016). However, here it is important to point out that although TRs in present study achieve high scores on measures of phonemic awareness, a significant proportion of the TR sample do not reach the performance on the maximum score, as has been suggested in the mono- and cross-linguistic Finnish studies. Like our study, Ziegler et al. (2010) included children in the second grade, i.e., in the early years of reading instruction, when it is still possible to detect strong effects of phonemic awareness. According to the same authors, the influence of phonemic awareness on reading development is more important before the onset of learning to read or in the early years of instruction than later, and the same variable is more prone to ceiling effects in transparent than in nontransparent orthographies (for a deeper discussion about phonemic awareness cross-linguistically, see Ziegler et al., 2010). Croatian is a language of transparent orthography, with some exceptions in the correspondence of graphemes and phonemes, but although participants scored well, a significant proportion of the TR sample in our study has not revealed a ceiling effect in phonemic awareness tasks. Therefore, we can say that the importance of phonemic awareness in the early years of learning to read is not only defined by the distinction between transparent and non-transparent orthography, but also by different degrees of transparency (or script entropy) in languages with transparent orthography. It may be that this small grapheme-phoneme inconsistency in Croatian prolongs the mastery of phonemic awareness. Further, it should also be mentioned that there are clear differences between the studies in the way they assess phonemic awareness. In some studies, such as that of Sánchez-Vincitore et al. (2022), second-grade children were only asked about initial phonemes, whereas Ziegler et al. (2010) asked children to delete initial phonemes in all languages except Finnish. These methodological differences in the way phonemic awareness was assessed certainly contributed to the different results, as different types of phonemic awareness require different degrees of implicit and explicit awareness.

Further, in the TR group, good phonemic awareness was supported by high scores on other phonological skills, such as the ability to temporarily store (PWM) and repeat phonological units without meaning, as well as to rapidly recall lexical units, while simultaneously decoding their phonological strings accurately (RAN). Children with reading difficulties had significantly lower scores on all these phonological variables, which implies that they are less equipped with the phonological skills required for decoding in the second grade.

However, it is important to note that, at the end of the second grade, TRs did not reach the peak on decoding tasks i.e., reading a list of words and pseudowords. Further analysis revealed that this group of participants exhibited lesser accuracy when reading pseudowords than words (see Table 3). This means that relying on a phonological route without any semantic support generates a huge cognitive load in the second grade, even for TRs. The average score of PRs for reading pseudowords was 6.73, suggesting that this group of participants could accurately read less than half of the pseudowords. The data obtained from TRs in the present study is not entirely consistent with previously published Finnish data i.e., with Torppa et al. (2007) statement that in learning to read in languages with highly regular orthography, the development of reading skills is very rapid after the beginning of reading instruction and that qualitative changes from a non-reader to an accurate decoder occur within short periods of time. Based on the data analyzed in the present study, one cannot argue that students in Croatian become good decoders after 2 years of formal instruction and in any case, there is still room for improvement in reading accuracy.

Moreover, reading speed variables show that PRs take significantly more time to decode words, pseudowords, and text. It is important to note that there is high variability in the time required for decoding in both groups, but the variability is much greater in the PR group.

In terms of language comprehension, data shows that TRs have a better understanding of spoken discourse than their PR peers. Good performance on all measures that constitute pre-reading skills, as well as on decoding and language comprehension, ensures good development of reading comprehension skills. Weaker performance of PRs on the same measures from the time they begin of learn to read results in a gap between pre-reading skills and reading comprehension.

### Association between decoding, language comprehension and reading comprehension

Examination of correlations in TR group showed that correlation between decoding and reading comprehension was not significant. As the TRs performed relatively well in decoding, a weakening of the association between decoding and reading comprehension is to be expected at this age. As the ability to decode is automated at this age in this group, language comprehension is related to reading comprehension. In PR group, both decoding and language comprehension were significantly correlated with reading comprehension, with the correlation between language and reading comprehension being stronger. Examining the correlations for these two groups separately indicates that, at the end of the second grade, decoding is important for reading comprehension only for the PRs, while language comprehension is important for reading comprehension for both groups.

As noted in Florit and Cain (2011) meta-analysis, the relationship between decoding and reading comprehension is very high in the early phases of reading development, but over time, the strength of this relationship diminishes as the typical child improves his or her decoding skills. These findings have been confirmed in many other mainly longitudinal studies. For example, Torppa et al. (2016) observed children from kindergarten to third grade and found a very weak relationship between these two constructs in the second and third grades. In contrast, when we observe children who have difficulty in reading, i.e., when reading processing is burdened with processing difficulties, it appears that some early proximal skills, in this case decoding, have an extended duration of action.

To investigate whether decoding and language comprehension predict reading comprehension in Croatian, we conducted linear regression analysis with reading comprehension as a criterion. The results of the regression analysis of the TR group showed that language comprehension (as a measure of listening comprehension), but not decoding (as a measure of accuracy), made a significant contribution to reading comprehension at the end of the second grade. On the other hand, results of the same analysis showed that both decoding and language comprehension contribute significantly to reading comprehension for PRs. These data suggest that TRs who have mastered decoding by the end of the second grade mostly rely on language comprehension to understand what they read, which is consistent with developmental expectations for reading (Florit and Cain, 2011). However, for PRs to achieve this complex cognitive ability, language comprehension alone is not sufficient to understand the text and hence they rely heavily on their decoding skills, which they have yet to master. This in turn supports the hypothesis that in the case of difficulties in reading processing, the reader draws on all his resources and activates all proximal skills, in this case both - those that are theoretically expected to be dominant skill at the end of the second grade and those that are no longer expected to be.

Additionally, decoding and language comprehension explained only 6% of the variance in reading comprehension of the TRs, resulting in an unsignificant model, while explaining 19% of variance in reading comprehension of the PR group at the end of the second grade. In other words, the total percentage of variance explained by the included variables is higher in the PR group, suggesting that these components still play a greater role in the condition when reading difficulties are present, while the role of the same variables in the TR group decreases. Since there is still a large percentage of variance that is not explained by these variables, it can be assumed that many other variables may have an influence on reading comprehension. These factors could be, for example, the child’s reading motivation and instructional methods. In their cross-linguistic study, Soodla et al. (2018) showed that children in Finland are more successful in comprehending what they read than their peers in Estonia. The authors stated that part of the explanation for these findings could be the child-centered instructional practices that are consistently implemented in all schools across Finland and in the nature of reading instruction, which is much more effective in Finland, largely because the teachers have good metacognitive knowledge about the instructional strategies they use.

### Profiles of reading difficulties in PRs

In order to examine the type of difficulties of PRs in detail, the group was divided into four subgroups based on decoding and language comprehension scores. Results showed that, based on a cut-off of −1 *SD* of the results of TRs, four heterogeneous profiles, i.e., subgroups of PRs with different proportions were identified in the second grade. The two most common groups were children with lower scores in decoding (31%), and children who had lower scores in both decoding and language comprehension (38%). Interestingly, very similar prevalence rates were reported in the study by Catts et al. (2003) who reported on proportion of children who had problems only with decoding and those who had problems with both decoding and language comprehension. Furthermore, our data are consistent with Tunmer and Greaney (2009) statement that mixed reading disabilities are the most common profile. These two groups of children – those with lower scores in decoding or lower scores in both decoding and language comprehension – who account for about 69% of all PRs, have a deficit in word recognition, the most important dimension for children to master in the early school years. Compared to the Finnish study (Torppa et al., 2007) that was able to identify only slow decoders (since PRs do not have a problem with accuracy in Finnish), in the present study, we observed children in the same grade level as in the Finnish study and identified students who achieved lower scores in decoding in comparison to TRs. This means that there were children among the PRs who had extremely low results on accuracy, indicating that for some children decoding might present a huge obstacle in learning to read in Croatian.

Our findings also show that only 14% of PRs achieved lower scores in language comprehension and relatively good scores in word recognition (i.e., indication for poor comprehension skills, Figure 2) when grouped based on TRs language comprehension scores. At the same time, a quarter of these with an indication for poor comprehension skills had extremely low scores (below −3*SD* on language comprehension compared to TR). This prevalence rate is also very similar to the one reported in Catts et al. (2003) for the group of poor comprehenders.

The last defined group is the one with the indication for non-specified difficulties, for which the results showed that 17% of PRs in our sample were classified in this group. In our more detailed analysis, two profiles were distinguished within this group - first, a somewhat larger group of slow decoders, and second, a somewhat smaller group with average results on most skills and with sporadic difficulties on different individual tasks. Due to these sporadic difficulties, it is important that the latter group continues to be monitored throughout schooling, whereas there are others who quite obviously slowly “grew out” of these difficulties.

Based on patterns of results we could distinguish five subgroups - those with lower scores primarily in accurate decoding (poor decoders), those with lower scores primarily in decoding speed (slow decoders), those with lower scores in both measures – decoding and language comprehension (mixed reading difficulty), those with lower scores on reading comprehension only (poor comprehenders), and group with sporadic difficulties. This profiling is very similar to the categories proposed by Torppa et al. (2007). Namely, based on an advanced mixture modelling procedure for two latent factors - one for word recognition fluency and one for reading comprehension and their covariance - measured at four time points in the first 2 years of school, Torppa et al. (2007) categorized three subtypes of reading difficulties - mixed reading difficulties, poor decoders, and poor comprehenders. The only difference between the profiles obtained in our study and the study of Torppa et al. (2007) is that our data also revealed the pattern of difficulty in accurate decoding. This result leads to the conclusion that it is important to look at decoding in terms of both accuracy and speed, and not just one of them. This is consistent with the double deficit hypothesis (Wolf and Bowers, 1999), which emphasizes the possibility of impairment in skills that primarily contribute to decoding accuracy such as phonological awareness, or impairment in skills that primarily contribute to decoding speed such as RAN, or both simultaneously.

This study showed that, among PRs, the highest proportion of children have problems with decoding (i.e., three out of five profile groups – poor decoding skill, mixed reading difficulties, and slow decoding skill). This means that teachers should be able to identify decoding difficulties relatively easily, more easily than comprehension difficulties, because there are more visible patterns that they can recognize and thus compare student performance. Therefore, approximately 78% of children should be screened in the second grade, rather than waiting until the third grade, which is the usual practice in Croatia, therefore prolonging their failure in reading because they lack additional educational support. This support can be provided in form of tiering, i.e., “a readiness-based instructional approach in which all students work with the same essential knowledge, understanding, and skill, but at different levels of difficulty based on their current proficiency with the ideas and skills” (Tomlinson and McTighe, 2006, p. 107). After providing first-tier services in the general education classroom, schools can offer the second-tier services provided by tutors in small groups. The last are third-tier services provided by intervention specialists in individualized settings. Unfortunately, in the Croatian educational system, there is currently no tier 2, but a direct transition from tier 1 to tier 3, which is sometimes very late.

### Limitations and further research

The results of the study must be considered in the light of a few limitations. As stated in the introduction, numerous non-language and non-cognitive skills (e.g., strategies and methods of reading instruction) have a significant impact on reading achievement. The internal characteristics of the child such as gender or the motivation to read, as well as external characteristics such as parental education, family socioeconomic status and family literacy have been reported to substantially impact reading development (e.g., Psyridou et al., 2021). For example, in Dutch studies van Bergen et al., 2011 and van Bergen et al., 2012), differences in parental education between children with and without dyslexia were similar to those found in the present study - parents of children without dyslexia had a higher level of education than parents of children with dyslexia. Therefore, the authors concluded that parental education could be an additional factor influencing children’s outcomes through heredity and the home literacy environment. Since those variables were not considered in this study, it would be worthwhile to include them in future work and examine their contribution to reading development.

In addition to these internal and external factors, it would also be worthwhile to include other linguistic and metalinguistic abilities and examine their influence on reading development. For example, since Croatian is a morphologically rich and complex language, it would be good to see to what extent and in what direction metamorphological awareness influences reading development. This will undoubtedly be one of the next studies, precisely because of the role that morphology plays in the Croatian spoken language (see Kovačević et al., 2009). As Croatian is a *prodrop language*, it can be assumed that this also has a major influence on reading development.

Moreover, this is the first time that reading development in the school years in Croatian has been approached systematically, which means that the authors of the paper were faced with the demanding assignment of creating reliable reading tasks. Because these tasks have not been previously verified, some of them, such as the oral language comprehension tasks, have somewhat less consistency, which may have affected our results. Also, data shows that on some tasks TR group had almost a maximum result, which probably indicates that those tasks are too easy. Since ceiling effects can significantly impact the results of an empirical study by reducing variation, distorting significance levels, and biasing effect sizes, the results obtained here should only be interpreted in accordance with the characteristics of the sample of this study. This is especially stands for the listening comprehension task since it was used for profiling of PR group, and restricted standard deviation of TR group has direct implications on it. Probably the results of profiling would be somewhat different if the used tasks were not so easy i.e., there was more variability in TR results, which would set the cut-off point of −1*SD* somewhat lower. However, it should be noted that PR group was identified based on different criteria (those available in Croatia at this moment), not on tasks used in this research, and that results of this group was also high on this task. Because of this, relatively high set cut-off point is in line with results of PR group in this research. Nevertheless, further research is needed for developing somewhat more difficult tasks that would enable better discrimination of TR and PR.

Another limitation is that this is a cross-sectional study, so it is not possible to draw conclusions about developmental characteristics. In this type of study, we can only determine the features of reading skills at a particular point in time, namely, at the end of the second grade. Moreover, we agree with Sánchez-Vincitore et al. (2022), where they stated that the best way to define predictors of reading development is through a longitudinal study. Longitudinal studies allow the investigation of changes in the contribution of each variable over time, which facilitates the description of developmental trajectories. In terms of classification of the reading subtypes, longitudinal follow-up studies allow verification of reading subtype stability. They also allow the observation of heterogeneity in growth, in contrast to cross-sectional studies, which only allow determination of heterogeneity in reading profiles in a specific unit of time. Therefore, it would certainly be important to base future studies on dependent samples of participants over a longer period of time. This is especially important for tracking profiles and determining whether and how they change from the early stages of learning to read to the later stages.

Obviously, a much larger number of participants should be included in the clinical group, especially if one wants to define profiles of difficulties (which leads to a significant reduction in the number per group of profiles). It is also necessary to develop standardized measures to assess reading competence and thus calculate precise threshold values. Nevertheless, the results of the present study point to the heterogeneity of the difficulty profile underlying PRs. A better understanding of the specifics of each profile will allow for reliable identification and improved designs for intervention programs in the educational system.

## Conclusion

Reading is an important academic skill and children who exhibit reading difficulties are more likely to experience various negative professional and personal consequences. In order to successfully identify these children as early as possible, one must first understand how reading skills are developed and what exactly describes the course of reading development in typical children and children who demonstrate reading difficulties from the beginning of formal reading instruction. Therefore, it is important to monitor the development of reading skills in terms of the increase in years of formal instruction. In this sense, the data collected in the present study can serve as a starting point for further studies in Croatian.

This study bears clear implications for education and clinical practice. As expected, TRs perform better than PRs on all underlying phonological and reading measures. It is important to point out that TRs score high but fail to reach the peak even on the underlying cognitive variables of decoding, such as measures of phonemic awareness. Failure to reach the peak, which is somewhat expected at this age for languages with transparent orthography, indicates subtle differences in transparency that should not be ignored. Further, language comprehension is a significant predictor of reading comprehension of Croatian TRs after 2 years of formal reading instruction, i.e., in the consolidated alphabetic phase. In addition to language comprehension, PRs still rely on decoding, i.e., decoding is still a significant predictor of reading comprehension in this group. This finding supports the hypothesis that, when the reading process is burdened with processing difficulties in any part of this skill, e.g., decoding accuracy, decoding speed, or reading comprehension, all proximal skills that support reading are activated, even those whose contribution is more important in the early stages of learning to read.

In the Croatian educational system, regarding learning to read, the *wait-to-fail* approach is widespread. Knowledge of reliable predictors of reading and understanding of the typical reading development can ensure early identification of PRs. Early identification promotes the development of early intervention programs, and as Solheim et al. (2021) highlighted, early identification followed by intensive support is the most effective solution for promoting the reading skills of PRs, especially before they have had extensive experience with failure. Our study profiled five different types of indication of reading difficulties, the most common one being a mixed reading difficulty. Three out of five profiles show decoding problems. Since decoding difficulties are associated with relatively easily noticeable manifestations (e.g., too many errors or extremely slow decoding), teachers should be able to recognize and observe decoding difficulties in children at an early stage of learning to read and respond proactively.

By focusing on early phases of reading instruction and analyzing what constitutes different aspects of reading skills, as well as by identifying and describing early reading profiles, our study outlines clear educational and clinical implications in the early school years. In order to obtain a clearer picture on developmental trajectories, the next steps would be to identify later reading profiles and to track changes in the children’s development over time in a longitudinal study.

## Data availability statement

The raw data supporting the conclusions of this article will be made available by the authors, without undue reservation.

## Ethics statement

The studies involving humans were approved by the Ministry of Science and Education of the Republic of Croatia (Class: 602-02/21-01/26; Number: 561-03-01/7-21-2; March 19, 2021). The studies were conducted in accordance with the local legislation and institutional requirements. Written informed consent for participation in this study was provided by the participants’ legal guardians/next of kin.

## Author contributions

JKK: Conceptualization, Methodology, Supervision, Writing – original draft, Writing – review and editing. NR: Data curation, Methodology, Writing – review and editing. VR: Data curation, Formal analysis, Methodology, Validation, Writing – review and editing. AMŠ: Writing – review and editing. ML: Conceptualization, Writing – review and editing. AŠ: Writing – review and editing.
