# Supplementary material for: Predictors of reading comprehension and profiling of poor readers in Croatian: educational and clinical perspectives
Source: Front Psychol. 2024 May 14;15:1297183. doi: 10.3389/fpsyg.2024.1297183 (PMC11135172; doi:10.3389/fpsyg.2024.1297183)
Supplement: Supplementary file 1 [file Table_1.DOCX]

**Appendix**

Table A1. Inclusion and exclusion criteria for participation in the study

| **Typical readers (TRs)** | | **Poor readers (PRs)** | |
| --- | --- | --- | --- |
| Inclusion criteria | Exclusion criteria | Inclusion criteria | Exclusion criteria |
| The student is enrolled in the second grade of the primary school for the first time. | The student is not enrolled in the second grade of primary school for the first time. | The student is enrolled in the second grade of the primary school for the first time. | The student is not enrolled in the second grade of primary school for the first time. |
| The student is enrolled in the same class as other children from a typical readers sample. | The student is enrolled in a regular classroom following an individualized education program with individualized procedures and/or content adaptation. | The student is enrolled in a regular classroom following a regular program or individualized education program with individualized procedures. | The student is enrolled in a regular classroom following an individualized education program with content adaptation. |
| The student is enrolled in a regular classroom following a regular program. | The student is enrolled in a special education classroom. |  | The student is enrolled in a special education classroom. |
|  | The student has been recognized as having reading difficulties based on the school Orientation list of difficulties^[[1]](#endnote-1)^ or diagnosed with dyslexia based on medical criteria. | The student has been recognized as having reading difficulties based on the school Orientation list of difficulties or diagnosed with dyslexia based on medical criteria. |  |
| Croatian is the student’s first language (L1). | Croatian is not the student’s L1. | Croatian is the student’s L1. | Croatian is not the student’s L1. |
| The student has normal hearing. | The student has impaired hearing. | The student has normal hearing. | The student has impaired hearing. |
| The student has normal visual processing (with or without glasses). | The student has impaired visual processing (with or without glasses). | The student has normal visual processing (with or without glasses). | The student has impaired visual processing (with or without glasses). |
| The student doesn't have any motor impairments. | The student has motor impairments. | The student doesn't have any motor impairments. | The student has motor impairments. |
| The student doesn't have any intellectual difficulties. | The student has intellectual difficulties. | The student doesn't have any intellectual difficulties. | The student has intellectual difficulties. |
| Psychological assessment revealed typical cognitive/ intellectual/ emotional functioning. | Psychological assessment revealed atypical cognitive/ intellectual/ emotional functioning. | Psychological assessment revealed typical cognitive/ intellectual/ emotional functioning. | Psychological assessment revealed atypical cognitive/ intellectual/ emotional functioning. |
| The student doesn't show any language, reading, writing, or speech fluency difficulties during assessment. | The student shows language, reading, writing, or speech fluency difficulties during assessment. | The student doesn't show any speech fluency difficulties during assessment. | The student shows speech fluency difficulties during assessment. |
| The student doesn't have unexpectedly low academic achievement. | The student has unexpectedly low academic achievement. | The student has unexpectedly low academic achievement. | The student doesn't have unexpectedly low academic achievement. |

Table A2. Variables on which children in the non-specified group had difficulties.

| **Participants in the non-specified group** | **Profile of difficulties** | | | | | | | |
| --- | --- | --- | --- | --- | --- | --- | --- | --- |
|  | PA- b/s | PA- d/a | PWM | RAN | RC | W-read. speed | PW-read. speed | Text-read. speed |
| **1** |  |  |  |  | 1 |  |  |  |
| **2** | 1 |  |  | 1 | 1 |  |  | 1 |
| **3** | 1 |  |  |  |  | 1 | 1 | 1 |
| **4** |  | 1 |  |  | 1 | 1 | 1 | 1 |
| **5** |  |  | 1 | 1 | 1 |  |  |  |
| **6** |  | 1 |  | 1 | 1 | 1 | 1 | 1 |
| **7** |  |  |  |  |  |  | 1 |  |
| **8** |  |  | 1 | 1 |  |  |  |  |
| **9** | 1 |  |  | 1 | 1 |  |  |  |
| **10** |  | 1 |  | 1 | 1 | 1 | 1 | 1 |
| **11** |  |  |  | 1 | 1 | 1 | 1 | 1 |
| **12** |  | 1 |  | 1 | 1 |  |  |  |
| **13** | 1 |  |  | 1 |  |  |  |  |
| **14** |  | 1 |  |  | 1 | 1 | 1 | 1 |
| **15** |  |  |  |  |  | 1 | 1 | 1 |
| **16** |  |  |  |  |  | 1 | 1 | 1 |

*Note: The presence of a certain difficulty is indicated in blue.*

Legend: PA-b/s – Phonemic awareness - blending and segmentation; PA-d/a – Phonemic awareness - deleting and adding; PWM – Phonological working memory; RAN – Rapid automatized naming; RC – Reading comprehension; W-read. speed – Word list reading speed; PW-read. speed – Pseudowords list reading speed; Text-read. speed – Text reading speed

1. The Orientation list of difficulties is an integral part of the Ordinance on primary and secondary education of children with developmental disabilities. It is used to define the type of a student's difficulties so that he or she can receive professional support appropriate to his or her needs. [↑](#endnote-ref-1)
